# On the etiology of aesthetic chills: a behavioral genetic study

**DOI:** 10.1038/s41598-022-07161-z

**Published:** 2022-02-28

**Authors:** Giacomo Bignardi, Rebecca Chamberlain, Sofieke T. Kevenaar, Zenab Tamimy, Dorret I. Boomsma

**Affiliations:** 1 Department of Language & Genetics, Max Planck Institute for Psycholinguistics, Nijmegen, The Netherlands; 2 Department of Biological Psychology, Netherlands Twin Register, Vrije Universiteit Amsterdam, Amsterdam, The Netherlands; 3 Department of Psychology, Goldsmiths University of London, London, UK; 4 Max Planck School of Cognition, Stephanstrasse 1a, Leipzig, Germany

**Keywords:** Behavioural genetics, Psychology

## Abstract

Aesthetic chills, broadly defined as a somatic marker of peak emotional-hedonic responses, are experienced by individuals across a variety of human cultures. Yet individuals vary widely in the propensity of feeling them. These individual differences have been studied in relation to demographics, personality, and neurobiological and physiological factors, but no study to date has explored the genetic etiological sources of variation. To partition genetic and environmental sources of variation in the propensity of feeling aesthetic chills, we fitted a biometrical genetic model to data from 14,127 twins (from 8995 pairs), collected by the Netherlands Twin Register. Both genetic and unique environmental factors accounted for variance in aesthetic chills, with heritability estimated at 0.36 ([0.33, 0.39] 95% CI). We found females more prone than males to report feeling aesthetic chills. However, a test for genotype x sex interaction did not show evidence that heritability differs between sexes. We thus show that the propensity of feeling aesthetic chills is not shaped by nurture alone, but it also reflects underlying genetic propensities.

## Introduction

Aesthetic chills^1^ are embodied emotional-hedonic responses evoked by, among others, experiences with music^2^, poetry^3^, videos^4^, beauty in nature or art^5^, or even by eloquent speeches^6^. They are frequently self-reported by individuals during peaks of hedonic^7–9^ and emotional experiences^2,10–14^, such as sadness and happiness^12,15^, being moved^14^, feeling touched^10^, and the sensation of awe^6^.

An extensive body of research has documented the physiological and neurobiological correlates of aesthetic chills, although sample sizes tend to be small. Chills occur with somatic manifestations (but see^16^), with participants reporting sidewise sensation of thrills in the upper dorsal part of the neck, or in the spine and back^5^, shivers down the spine^17^, tingling sensations in the arm^18^, and more general diffuse bodily reactions^10^. Bodily reactions that are associated consistently across studies with concurrent dynamic peripheral changes, mainly by increases in phasic skin conductance, and changes in heart rate, and less consistently with drop in temperature or increase in respiratory rate, occur before, during and after chill onset^2,8,9,13,19–23^. Besides being associated with self-reported and somatic manifestations, aesthetic chills usually correlate with activity in brain regions that play a role in the representation of visceral and somatic states^24^, such as the bilateral insula (Ins)^3,7,25^ and the anterior cingulate cortex^7^, as well with several other brain regions that overlap with general reward mechanisms in the basal ganglia^3,7,9,25^, and in the orbitofrontal cortex^7^, plus other areas such as the supplementary motor area^7,25^, the thalamus, and the cerebellum^7,22,25^.

While most humans across cultures seem to have the capacity to experience aesthetic chills^1^, individuals vary widely in the intensity and frequency with which they experience them^2,6,26,27^. To date there is some evidence that demographic, personality and neurophysiological differences can account for some of this variation. For example, one study reports older individuals are more prone to report chills than younger ones^28^, and others suggest females are more prone to report chills than males^15,29^. It is worth noting however that results on the association between demographic factors are inconsistent across studies, with the vast majority reporting no significant effects of age^21,30^ or sex^2,21,31–33^.

Additional explanations for individual differences in the propensity of feeling chills come from studies on personality differences. Individuals who score higher on Openness to Experience (OE) tend to experience more chills, as measured both by self-report^26,27,33–35^ and physiological measures^26^. However, as for demographic correlates, it is also worth noting that the consensus on the relationship between personality factors and chills is far from being unanimous, with some suggesting higher correlations between emotional-aesthetic components of OE and the propensity of feeling chills^33^, and others suggesting cognitive components of OE to play a bigger role^26^. The picture is further complicated by few studies that suggest other aspects of personality play a role too^4,13,21^, but, contrary to the association with OE, such studies have rarely been replicated.

Variation in the frequency of experiencing chills has also been accounted for by functional brain differences. For example, data obtained from resting-state functional Magnetic Resonance Imaging (rs-fMRI) on 1000 subjects indicates that individuals more prone to experiencing chills have enhanced connectivity between the network composed by the Ins and the cinglulate cortex (anterior and posterior cingo-opercular network) with ventral default network, by the latter network with the anterior visual and the posterior temporal networks, by the lateral and the dorsal default networks, and by a decrease in functional connectivity between the cerebellum and the somatomotor cortex^36^. Further, preliminary evidence obtained on small samples of individuals, suggests that the individual tendency of experiencing chills also correlates with structural brain differences, such as higher tract volume between the superior temporal gyrus and both the anterior insula and the medial prefrontal cortex^20^, and resting physiological arousal, such as higher resting state skin conductance level^30^.

Beyond physiological correlates of chills, several psychological correlates, such as empathy^31^, modulation of negative affect^21^, and stress-related growth^37^ have been found in the past. For example, Johns et al.^37^ recently highlighted, in a moderately large sample, the relationship between proneness to feel aesthetic chills to the tendency of approaching stressful events in a way that facilitates positive growth.

The etiological sources of variation in aesthetic chills, i.e., how much of the observed variation can be explained by genetic and environmental factors, is yet unknown. Such lack of knowledge about the etiology is not unique to aesthetic chills alone but is shared among many studies on aesthetics. To our knowledge, there are only a few empirical investigations addressing the etiological sources of variation underlying individual differences in aesthetic experiences/appraisal^38–43^, many of which specifically focused on narrow aspects of aesthetics (e.g., preferences). These studies applied the Classical Twin Model (CTM) to distinguish genetic from environmental influences. Two studies from the 1970’s (Barron^38,39^) focused on individual differences in aesthetic sensitivity –defined by the authors as the extent to which one individual’s aesthetic judgment is in line with the opinion of experts- for paintings and drawings in a small sample of twins. The authors found contradicting results, with non-trivial heritability estimates ranging from 55 to 67% in the first study^39^, and trivial estimates in the latter^38^. Most recent studies from Zietsch et al.^43^, Germine et al.^41^, and Sutherland et al.^42^ focused on aesthetic preferences for faces. Heritability estimates were 33% for specific preferences for dimorphic male traits^43^, and 22–30% for more general individual preferences for faces respectively. The only study we are aware of going beyond preferences, from Butkovic et al.^40^, found 40% of individual differences in flow proneness from music—a subjective, pleasurable, and fully absorbing experience—to be explained by genetic factors.

Here we aim to investigate whether genetic effects can account for individual differences in the propensity of feeling aesthetic chills. To partition genetic and environmental sources of variation, we fitted a biometric genetic model to twin data, exploring a genotype by sex interaction by testing for both quantitative and qualitative sex differences. This allowed to test for differences in the importance of genetic influences on aesthetic chills and to test whether the same genes are expressed in men and women. We analyzed Item 43 of OE, “Sometimes when I am reading poetry or looking at a work of art, I feel a chill or wave of excitement”^44^ as a proxy for the propensity of feeling chills. This item was selected, because explicitly asking individuals if they feel chills is a good indicator of actual experienced chills measured in experimental settings^11,25,26^. For example, Colver and El-Alayli^26^ found the subjective reports of the experience of chills correlate with chills measured by a combination of button presses and galvanic skin responses (*r* = 0.90). Kleipzig et al.^25^ found a modest correlation between self-reports of how often individuals experience shivers and report chills while undergoing fMRI (*r* = 0.63). Further, Item 43 seems capable of tapping into individual differences which are highly shared among cultures, being the first or the second best predictor of OE^1^; to significantly correlate (*r* = 0.29) with chills measured by the co-occurrence of self-reported feelings and physiological changes^26^; and to capture neurophysiological differences between individuals^36^, by being significantly associated (0.09 $\le r\le$ 0.11) with the strength of the functional connections between different resting state networks.

## Methods

### Participants

The data were obtained from the Netherlands Twin Register (NTR), a longitudinal cohort established in 1987 by the department of Biological Psychology at the Vrije Universiteit Amsterdam. Data on the self-reported propensity of feeling aesthetic chills were collected by mailed surveys (see^45^ for details), collected in 2004 (NTR survey 7^46^) on 6760, in 2009 (NTR survey 8^47^) on 10,176 and in 2013 on 9419 twins (survey 10^48^). In all surveys, participant consent was obtained (after reading the consent form and study information, our institutional review board consider returning the survey as providing consent). Written informed consent was obtained from individuals when volunteering to become a participant in the NTR. The studies were in accordance with the declaration of Helsinki and were approved by the Central Ethics Committee on Research Involving Human Subjects of the VU University Medical Centre, Amsterdam, an Institutional Review Board certified by the U.S. Office of Human Research Protections (IRB number IRB00002991 under Federal-wide Assurance- FWA00017598; IRB/institute codes, NTR 03-180). For ANTR Survey 7: METC 03/181; for ANTR Survey 8: METC 2008/244; for ANTR Survey 10: METC 2012/433. After excluding 320 pairs, for which no data for the item 43 were available across surveys, and data from 5 twin pairs due to missing information on age for both twins, we analyzed data on 14,127 twins (9466 females), ranging from 14 to 97 years old, with mean age = 30 (SD = 13). Our procedure for data selection when multiple surveys had been completed, is detailed below. Table 1 shows the numbers of twins and the number of complete pairs (i.e., pairs in which both twins completed each survey) or incomplete pairs (i.e., pairs for which only one of the two twins completed the survey). Zygosity in same-sex pairs was based on genotyping for part of the sample and on survey information for others. In adult NTR participants, multiple surveys contain zygosity items (five items about resemblance and three items about confusion by parents by parents and others) which were answered by the twins. In considering how well these items assess zygosity, we only included data from same-sex twins whose survey information had been completed before they received the results of their DNA tests. This resulted in a sample of 3512 twins. 60% of the data were randomly assigned to be in a training set and 40% in a testing set. The first available survey with zygosity items was originally analysed^45^. The outcomes of linear discriminant analysis of the training set were used to predict zygosity in the testing set (N = 1362). Zygosity prediction based on survey items was accurate in 95.9%. The item that distinguished best between MZ and DZ twins was “Were you each other’s spitting image as children?” (see *Appendix. Zygosity* in^45^ for details.) Test-reliability correlations for the twins who have completed more than one survey (N = 6923) were calculated for males and females separately. To avoid confounding effects due to familiar resemblances, data from one randomly selected twin per family were analyzed.

**Table 1:** Sample (N) of monozygotic (MZ) and Dizygotic (DZ) twin pairs per survey.

Survey	N pairs	MZ male	MZ female	DZ male	DZ female	DZ opposite sex
Survey 7	6195	931	2463	478	1137	1186
Survey 8	9100	1239	3242	747	1684	2188
Survey 10	8302	1161	2935	663	1505	2038
Combined	**8995 (5132)**	**1279 (780)**	**2809 (2010)**	**813 (392)**	**1645 (914)**	**2449 (1036)**

Combined sample is shown in bold. Number of complete pairs is shown between parentheses.

### Materials

Self-report of chills was obtained from the short version of the NEO-personality Five Factor Inventory (NEO-FFI^44^). The NEO-FFI consists of 60 items rated on a five-point scale (1–5, strongly disagree, disagree, neutral, agree and strongly agree). The Openness to Experience (OE) scale contains an item (43) “Sometimes when I am reading poetry or looking at a work of art, I feel a chill or wave of excitement” which was selected as a proxy for the propensity of experiencing aesthetic chills.

### Procedures

For 49% of participants, we had more than one survey. To maximize sample size, data from survey 7, 8 and 10 were merged into one data file by randomized selection of twin pairs per survey. The selection of pairs followed a number of criteria: (1) we prioritized complete answers from twin pairs, i.e., twin pairs were selected when both twins reported scores for the Item 43 on one of the surveys; (2) if one of the two twin’s response was missing for all three surveys, we randomly selected a survey with the response for the other twin; (3) if twins took part in different surveys, we randomly selected data for the pair from one of the complete surveys. Of the reported combined sample size, 5132 were twin pairs who both completed the survey, of which 2790 were MZ, 1306 were same-sex DZ (DZss), and 1036 were opposite-sex DZ (DZos) twin pairs.

### Genetic analysis

We analyzed the data using the CTM to estimate the proportion of variance explained by genetic and environmental factors. Within the CTM, the observed phenotypic variance (P) can be decomposed into additive genetic (A), dominance genetic (D), common environmental (C), or unique environmental (E) components^49^. The A component captures all additive effects of alleles across genetic loci, while the D component captures non-additive and interactive effects of alleles at contributing genetic loci. For the environmental components, the C captures all environmental factors that are shared between twins. When twins are raised together, home-environment effects can be captured by C. The E component captures all factors that are non-shared between twins nor explained by genetic factors. Thus, the E component captures all the unexplained unsystematic variance, comprising measurement errors.

The decomposition of the variance is possible because different genetic association exist for MZ and DZ twins. Since MZ twins derive from the same fertilized egg their genetic material is ~ 100% shared (but see^50,51^). MZ twins thus share 100% of both additive as well as dominance genetic effects. DZ twins, on the other hand, derive from two fertilized eggs and share only 50% on average of the additive genetic effects, and only 25% of the dominance genetic effects (see^52^). Thus, within the CTM, the correlation between the A component within MZ twin pairs is equal to 1, while within DZ twins is equal to 0.5. Similarly, the correlation between the D component within MZ twin pairs is also 1, while for DZ pairs is 0.25. Further, since the C component captures all shared environmental effects and the E component captures all non-shared effects, the correlation for both MZ and DZ twins are set to 1 for the C component and 0 for the E component. As a consequence of these premises, when MZ twins resemble each other more than DZ twins on a given trait the heritability of such trait is considered to be non-trivial. Here, it is important to note that a model with four components cannot be statistically identified within the CTM, given that there is only enough information to estimate three components. Therefore only A, C and E or A, D, and E components can be estimated simultaneously^52^. A rule of thumb is to assess the twin correlations and fit an ACE model accordingly when the MZ correlation is not larger than twice the DZ correlation. In comparison, an ADE model is fitted when the MZ correlation is larger than double the DZ correlation. Components within a model (e.g., ACE) are then dropped (e.g., dropping C) to assess whether they contribute to phenotypic variation. If the model with fewer components (e.g., AE) fits the data as equally well as the model with more components, then the dropped component is evaluated to be not explaining any significant phenotypic variance.

To investigate sex differences in the etiology of the trait, we look into the difference in correlations between female, male, and opposite-sex twins. When the genotype affects phenotypic variation to the same extent in men and women (no quantitative sex differences), we expect MZ male-male correlations to be equal to MZ female-female correlations and DZ male-male, and female-female correlations also to be similar. When there is no difference in the expression of the genes in men and women (no qualitative sex differences), we expect that the correlation in same-sex DZ twins is equal to that of opposite-sex DZ twins. Under the assumption of no sex effects, the amount of phenotypic variance is expected to be accounted for by a similar amount of A, D, C, and E, and similar genes are expected to influence phenotypic variation in both sexes^53^.

The models were specified in OpenMx version 2.17.2^54–56^, in R-Studio, R version 3.6.2. Significance of the covariate (age), birth order effect and mean differences across zygosity were tested by a series of models nested inside the saturated model. The goodness of fit of the model was evaluated by 1) likelihood ratio, that is by the difference in minus twice the value of the log-likelihood (− 2LL) between the two models, which has a *χ*^2^ distribution, with the degrees of freedom equal to the difference in the number of parameters, and by 2) the Akaike’s Information Criterion (AIC), by keeping the model with the lowest AIC as the best fitting model.

Genotype x sex interaction was tested for both quantitative sex differences (i.e., is the amount of variance in the propensity of feeling aesthetic chills accounted for by the same genetic effects across sexes?) and qualitative sex differences (i.e., are genes influencing variation in the propensity of feeling aesthetic chills the same in females and males?) in etiology. First, similarly to Vink et al.^53^, the significance of differences in means, variances, and covariances across sexes was tested by a series of models nested inside the saturated model. A sex-limitation model was evaluated to examine whether quantitative sources of etiological variation statistically differed between the sexes by constraining variance components to be equal for men and women. The goodness of the fit and the significance of the sub-models with variance components constrained to be equal across sexes were compared to the full model. Qualitative sex differences were tested by allowing genetic correlations (r_g_) between DZos to be freely estimated. The goodness of the fit and the significance were obtained by comparing the more parsimonious model in which r_g_ within DZos was constrained to 0.5. Mean, standard deviations, 95% CI, and within twin pair correlations were estimated in a saturated model. A variance decomposition model was compared to the saturated model. Subsequently, we test nested models, which were obtained by constraining one of the genetic or environmental variance components to zero. Heritability estimates were obtained as the proportion of genetic variance over the phenotypic variance.

## Results

### Descriptive statistics

Test–retest reliability was obtained for data from surveys 7 and 8 (5 years apart), surveys 8 and 10 (4 years apart), and surveys 7 and 10 (9 years apart). Single-item reliability estimates range from *r*(1488) = 0.58 ([0.54,0.61] 95% CI) and *r*(578) = 0.61 ([0.56,0.66] 95% CI) between survey 7 and survey 8 for female and male respectively, *r*(2118) = 0.58 ([0.55,0.60] 95% CI) and *r*(830) = 0.52 ([0.47,0.57] 95% CI) between survey 8 and survey 10 for female and male respectively, and *r*(1078) = 0.58 ([0.52,0.60] 95% CI) and *r*(410) = 0.51 ([0.44,0.58] 95% CI) between survey 7 and survey 10 for female and male respectively (all *p* < 0.001, after Bonferroni correction).

**Figure 1:**
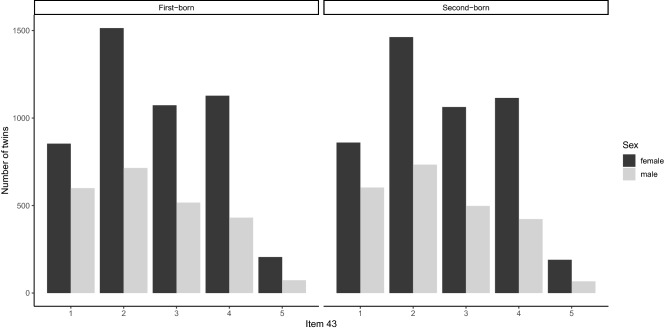
Distribution of item 43 “Sometimes when I am reading poetry or looking at a work of art, I feel a chill or wave of excitement”. The left panel shows the distribution for the first-born twin of the pair from female and male respectively. The right panel shows the distribution for the second-born twin of the pair from female and male respectively.

The distribution of the item 43 scores is given in Fig. 1 for first and second born twins, separately by sex. Individuals scale point frequency on the 5 Likert-scale ranged from 21% (“strongly disagree”) to 4% (“strongly agree”), with the majority of individuals (75%) distributed within the three central scale points. The finding that 21% of the firstborn twins do not report aesthetic chills in the combined survey is in line with some previous random-population sampling studies on chills^2,5,17,18,32^.

### Biometric modelling

**Figure 2:**
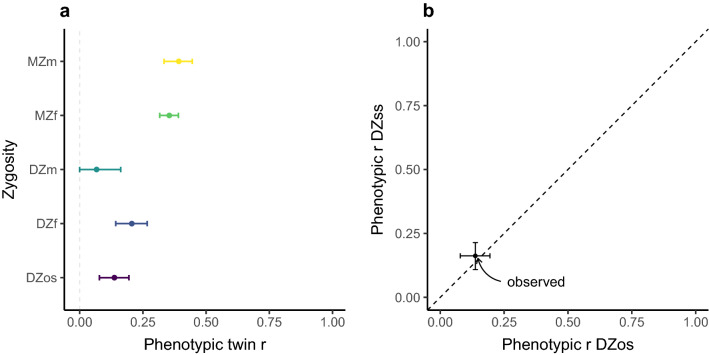
Phenotypic twin correlations. (**a**) Correlations (r) within twin pairs, error bars represent 95% CI. (**b**) Modified from Vink et al.^53^. The dashed line represents the expected slope for the relationship between DZss and DZos r when genotype x sex interaction effects on phenotypic variation are not present. The dot represents the observed DZos pair correlation versus the DZss pair correlation, extracted from the Sex:DZss same covariance model. The horizontal and vertical error bars represent the 95% CI for the DZos and the DZss 95% CI. *MZ* monozygotic, *DZ* dizygotic, *m* male, *f* female, *ss* same-sex, *os* opposite-sex.

Twin correlations from the saturated model, were *r* = 0.39 ([0.33,0.44] 95% CI) and *r* = 0.35 ([0.32,0.39] 95% CI) for MZ male and MZ female respectively, *r* = 0.07 ([0.00,0.16] 95% CI) and *r* = 0.21 ([0.14,0.27] 95% CI) for DZ male and DZ female respectively, and *r* = 0.14 ([0.08,0.19] 95% CI) for DZos (also see Fig. 2a). These correlations and CI suggested that an AE model to be most appropriate for describing these data. Table 2 shows the goodness of the fit comparison with the full saturated model. One the one hand, removing age as a covariate resulted in a deterioration of the model fit (-2LL = *χ*^2^(1) = 255.07, p < 0.001). On the other, removing birth order and subsequently zygosity mean and variance differences did not deteriorate the overall model fit (all p ≥ 0.80), indicating that mean and variance were not different across the first and the second-born and across zygosity. As expected, constraining mean scores to be equal across sexes resulted in a deterioration of the fit (− 2LL = χ^2^(17) = 113.68, p < 0.001), However, constraining variance to be equal across sexes, as well as constraining covariance to be equal across DZss and DZos, did not deteriorate the overall fit of the model (− 2LL = *χ*^2^(17) = 6.64, p = 0.99 and − 2LL = χ^2^(20) = 13.93, p = 0.83, respectively).

**Table 2:** Saturated model: Model-fitting results from five groups (MZ male, MZ female, DZ male, DZ female, DZos) model.

Model	-2LL	df	*χ*^2^	Δdf	p	AIC
Saturated model	43,163.60	14,101	NA	–	–	14,961.60
**Covariate**						
No age	*43,418.67*	*14,102*	*255.07*	*1*	< *0.001*	*15,214.67*
**Birth order**						
Same mean	43,165.21	14,105	1.61	4	0.80	14,955.21
Same mean and variance	43,167.16	14,109	3.55	8	0.89	14,949.16
**Zygosity**						
Same mean	43,169.35	14,113	5.75	12	0.92	14,943.35
Same mean and variance	43,170.22	14,117	6.62	16	0.98	14,936.22
**Sex**						
Same mean	*43,277.28*	*14,118*	*113.68*	*17*	< *0.001*	*15,041.28*
Same variance	43,170.24	14,118	6.64	17	0.99	14,934.24
MZ same covariance	43,171.43	14,119	7.823	18	0.98	14,933.43
DZss same covariance	43,177.14	14,120	13.54	19	0.81	14,937.14
DZ same covariance	**43,177.53**	**14,121**	**13.93**	**20**	**0.83**	**14,935.53**

In bold best-fitting model. In Italics models that showed deterioration of the fit. Models are reclusively nested starting from the most parsimonious model. For example, the “‘Birth order: same mean and variance model’ is nested from the most parsimonious ‘Birth order: Same mean’, while ‘Birth order: Same mean’ is not nested in the ‘Covariate: *no age*’ model, since removing the covariate results in a deterioration of the overall fit.

*MZ* monozygotic, *DZ* dizygotic, *ss* same-sex, *os* opposite-sex.

Phenotypic correlations for DZss and DZos, extracted from the model in which covariance across sexes were constrained to be equal (Sex:DZss same covariance model), were *r* = 0.16 ([0.11,0.21] 95% CI) and *r* = 0.14 ([0.08,0.19] 95% CI) respectively. The pattern of correlations between DZss and DZos indicates an absence of evidence of genotype x sex interaction effects (see Fig. 2b). Table 3 shows the results for the sex limitation models. The full AE model was fitted to data from males and females, with separate estimates for means and variance components. As expected, the mean scale point for item 43 was found to differ across sexes (− 2LL = *χ*^2^(1) = 106.32, *p* =  < 0.001). However, constraining DZos r_g_ to be equal to 0.5 did not deteriorate the model fit (− 2LL = *χ*^2^(1) = 2.16, *p* = 0.14). This indicated etiological sources of variation to not qualitatively differ across sexes. Moreover, constraining variance components across sexes to be equal did not deteriorate the model fit (− 2LL = *χ*^2^(3) = 2.19, *p* = 0.53). This indicated etiological sources of variation to not quantitatively differ across sexes either.

**Table 3:** Biometric model: model-fitting results for the sex limitation and the univariate models.

Model	minus2LL	df	*χ*^2^	Δdf	p	AIC	h^2^	c^2^	e^2^
**Sex limitation model**									
AE	43,177.56	14,119	–	–	–	14,939.56	–	–	–
AE: mean	*43,282.44*	*14,120*	*104.88*	*1*	< *0.001*	*15,042.44*	–	–	–
AE: DZos r_g_	43,179.72	14,120	2.16	1	0.14	14,939.72	–	–	–
AE: variance	**43,179.75**	**14,122**	**2.19**	**3**	**0.53**	**14,935.75**	–	–	–
**Univariate biometric model fitting**									
Saturated model	43,163.60	14,101	–	–	–	14,961.60	–	–	–
ACE	43,179.75	14,121	16.14	20	0.71	14,937.75	.36	.00	.64
AE	**43,179.75**	**14,122**	** < 0.001**	**1**	** > 0.999**	**14,935.75**	**.36**	–	**.64**
CE	*43,222.81*	*14,122*	*43.07*	*1*	< *0.001*	*14,978.81*	–	*.30*	*.70*
E	*43,621.39*	*14,123*	*441.64*	*2*	< *0.001*	*15,375.39*	–	–	*1*

In bold best-fitting models. In Italics models that showed deterioration of the fit. h^2^ heritability estimate; c^2^ shared systematic environment estimate, e^2^ unique unsystematic environmental estimate. The expected AE sex limitation model and the full univariate ACE model are tested against the full saturated model. Nested sex limitation and univariate models are tested against the expected AE and the full ACE respectively.

Phenotypic correlations, obtained from the most parsimonious model Table 2, were *r* = 0.37 ([0.33, 0.40] 95% CI) within MZ and *r* = 0.15 ([0.11,0.19] 95% CI) within DZ twin pairs. Phenotypic correlations suggested once more the AE model as the most appropriate model to describe the data.

**Figure 3:**
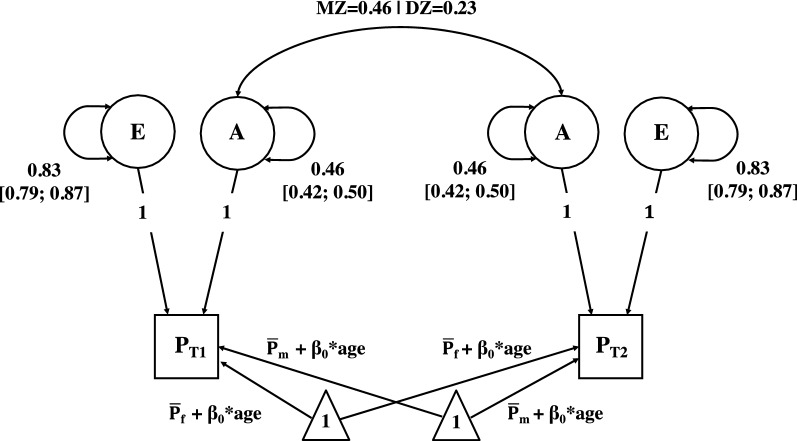
Best fitting AE biometric model. Final model with parameters $\overline{\mathrm{P} }$_f_, $\overline{\mathrm{P} }$_m,_ β_0,_ and latent factors A and E. The Squares represent twin (T) 1 and 2 observed phenotypes (P) in aesthetic chills. The triangle represents the mean estimates. The circles represent the additive genetic (A) and the environmental (E) factors, with their associated (unstandardized) variance. The arrows pointing to the square represent the genetic and environmental path coefficients. These were constrained at 1 and the variance of the latent factors was estimated. The double arrows across the variance component A represent the expected covariance within MZ and within DZ twin pairs. $\overline{\mathrm{P} }$ = grand means for females (f;$\overline{\mathrm{P} }$_f_ = 2.25) or males (m; $\overline{\mathrm{P} }$_m_ = 2.03); the regression for age is *β*_*age*_ = 0.01.

The final genetic univariate model fitting results and comparison are presented in Table 3. Constraining variance components A, and A and C to zero respectively deteriorated the model fit (all *p* ≤ 0.001). As expected, the final model AE (Fig. 3), with mean estimates adjusted for age (*β*_*age*_ = 0.01) equal to 2.03 for males and 2.25 for females (SD = 1.13), was the most parsimonious well-fitting one (− 2LL = *χ*2(1) ≤ 0.001, *p* ≥ 0.999). As shown in Table 3, the heritability estimate for the propensity of feeling chills is 36% (A = 0.36 [0.33, 0.39] 95% CI), while the remaining 64% of the phenotypic variance (E = 0.64 [0.61, 0.67] 95% CI) can be accounted for by unsystematic effects, such as environmental experience unique to the individual and measurement errors.

## Discussion

This research investigates and reports genetic and environmental sources of variation for the propensity of feeling aesthetic chills. We analyzed the variance of the NEO-FFI Item 43: “Sometimes when I am reading poetry or looking at a work of art, I feel a chill or wave of excitement”, which is a proxy measurement for the propensity of feeling aesthetic chills. We found that 36% of the variance in feeling aesthetic chills can be explained by additive genetic factors and the remaining 64% by environmental sources of variation. If we consider these results in the view of the test–retest reliability, which we obtained for data from surveys 4, 5 and 9 years apart, which ranged from around 0.51–0.61, the environmental variance reflects to a substantial extent reliable non-genetic causes of individual differences.

A lack of shared environmental sources of variation in the propensity of feeling chills should not come as a surprise, given that variation in most of the psychological human traits investigated so far show no relationship with shared environmental effects^57^. Moreover, we confirmed, in the largest sample used to investigate the role of demographic factors on chills to date, that females and older individuals are more prone than males and younger individuals to experience aesthetic chills. This is in line with some of the results previously obtained from the general population^15,28,29^. Additionally, it is also important to note that the directionality of the sex effect as found in this study was also consistent, as far as we know, with all of the studies that reported non-significant trends toward females reporting more frequent chills than males^2,31,33^.

We would like to note that, although apparently consistent with the separation call phenomena proposed by Panksepp^15^, our results are only partially supporting his hypothesis. Panksepp argued that chills are a genetically influenced trait that “resonate with ancient emotional circuits that establish internal social values”^15^, and that they probably evolved over time from a need for physical closeness induced by the separation between a mother and her infant. Such mother-infant relationships should have produced an enhanced selective propensity of experiencing chills in females. Our findings that variation in chills is influenced by genetic factors, and that females are more prone to experience chills, therefore partially support this hypothesis. However, our findings that no qualitative, nor quantitative, genotype x sex interaction effects affect variation in propensity on aesthetic chills do not support the separation-call hypothesis. Indeed, in line with results on a majority of other human traits^53,58^, our results are consistent with the hypothesis that the same genetic sources of variation in males and females influence variation in the propensity of feeling aesthetic chills.

We believe our results bring into focus questions that go beyond the descriptive nature of this study. Williams et al.^36^ argued that the enhanced connectivity between sensory and salience/default networks as found in their study, based on resting-state fMRI data of 1000 subjects from the Human Connectome Project (HCP), indicates that individuals who are more prone to experience chills from art and poetry, as measured by the item 43, are also individuals that can better integrate sensory information with internal emotional experiences. Moreover, preliminary evidence on a small sample recently indicated that augmenting sensory signals that mimic the physical experience of aesthetic chills can enhance individual social-affective cognition (e.g., empathy and pleasure;^59^).

Yet, how can one reinterpret such results in light of our present findings? Is it environmental exposure over one’s own lifetime to art and poetry that is causally shaping the connectivity as seen in Williams et al.^36^ or is it more a priori predisposition that makes individuals better at integrating sensory information with their internal states, or alternatively more sensitive to bottom-up somatosensory signals, that makes them more likely to reach such peaks of emotional-hedonic experiences? Clearly, further genetic informative studies, such as cross-trait cross-twin studies displaying both proneness of feeling chills and, for example, functional or structural brain metrics, are needed to answer these questions. However, our finding that approximately one-third of the variance in the propensity of feeling chills can be explained by genetic influences sheds some light on such questions.

Further, it is important to consider the limitations of our study. As other studies investigating complex traits in large samples^60,61^, we were constrained by what measurements were available. This reduced the complexity of the variation in the propensity to feel aesthetic chills to variation in a single item. Additionally, it is worth noting that the NEO-FFI item 43 explicitly asks for the propensity of feeling chills, or wave of excitement, only from art or poetry. As such, Item 43 neglects the possibility of drawing any distinction between different types of chills^25,62,63^ and omits many other possible causes of chills. This limited our capacity to obtain a more nuanced estimate of aesthetic chills. For example, although a previous study found the item 43 to be correlated with the number of chills experienced from music^26^, further studies are still needed to claim that etiological sources of variation influence the propensity of feeling chills from music specifically. Moreover, even though the test–retest reliability for the item 43 may be considered good for a single item, it was not perfect. However, if this had any impact on our results it was through increasing the measurement error, which by definition is included in the estimate for the unsystematic environmental sources of variation. As such, our estimates on the effect of additive genetic variation should be considered to be, at worst, a lower bound for the real effect of genetic influence on the propensity of aesthetic chills. Another consideration is that the study of the etiology of a trait detached from any personality background represents a rather unorthodox approach to etiological studies. Our approach is motivated by the specific interest to what item 43, as highlighted above, pertains to be, that is, variation in the proneness to aesthetic chills and not by its relative weight on OE. Although not common, such an approach (i.e., biologically informative analysis at the item level) has found support in recent literature^36,60,64^. For example, Franic et al.^64^ showed, in a partially overlapping sample to the one used in this study, the role of independent genetic contribution on the variation of single items by finding evidence against the hypothesis of personality factors as full mediators of genetic effects. Further, Williams et al.^36^, while exploring the association of Item 43 with the strength of the functional connections between different resting-state networks, found that an item being part of a broader personality factor can be more strongly associated with variability in a biological trait of interest than a personality factor itself.

Finally, it is important to note that it would be premature to reach any conclusions regarding the putative mechanisms underlying the genetic factors influencing aesthetic chills on the basis of the findings of this study alone. Some theoretical work makes predictions regarding biological mechanisms that might contribute to the experience of aesthetic chills. For example, under the knowledge-instinct theoretical perspective, positive chills emerge when the similarity between sensory information and the a priori mental representation of such information is maximized, a phenomenon that has been attributed to the modulation of the activity in the neural circuitry playing a role in the experience of chills, mainly via opioidergic or/and dopaminergic transmission^65^ (with contradictory evidence either supporting^5^ or not supporting the first^66^ and more consistent evidence supporting the second^9,67^, respectively). It would be tempting to speculate on the role that genes coding for proteins playing in such neuromodulatory mechanisms have on the genetic variation as found in this research. However, the recent past taught us that heritability estimates from twin studies are usually explained by thousands, if not hundreds of thousands of tiny genetic effects, even for traits for which the causal chain from genes to behavior seems to be shorter than expected (see for example^68,69^).We thus warn against any speculation on single putative causes, and thus call for further genetic informative studies. Genome wide association and functional annotation studies would be especially useful in exploring the whole genome in order to inspect which genetic variations can be associated with variation in the propensity of aesthetic chills, and to inspect whether genetic variations associated with the propensity of feeling chills are enriched in brain tissue or elsewhere in the body. Further, although our study focused solely on the propensity of feeling aesthetic chills, its relationship with any other item is not uninteresting per se. For example, item 43 shares its etiology with other OE (e.g., “Intrigued by patterns in art and nature”) and non-OE (e.g., “Goes to pieces under stress” and reverse coded “Rarely fearful or anxious”, “Seldom sad or depressed”) personality items, making it an interesting candidate for further descriptive associations studies.

## Conclusion

Aesthetic chills are a somatic marker of peak emotional hedonic responses. Previous inconsistent evidence suggested that demographic, and somewhat more consistent evidence has suggested that personality and neurobiological factors, can account for part of the observed variation in feeling chills. Here, we confirmed that females are more prone than males, even if to a small degree, to experience aesthetic chills and that older individuals tend to report experiencing chills more than younger ones. Critically, we revealed that genetics play a role in the individual differences in the propensity of feeling chills, thus indicating that the tendency of experiencing hedonic peaks of emotional reactions to art and poetry is not shaped by nurture alone, but is also influenced by genetic predispositions, as thousands of other human traits are^57^.

## Data Availability

The data that support the findings of this study are available from Netherlands Twin Register (NTR; www.tweelingenregister.org) but restrictions apply to the availability of these data, which were used under license for the current study, and so are not publicly available. Readers interested in having access to the data can consult Ligthart et al.^45^. The code used to analyze the data is available at https://github.com/giacomobignardi/h2-aesthetic-chills.
